# Markers of epithelial-to-mesenchymal transition reflect tumor biology according to patient age and Gleason score in prostate cancer

**DOI:** 10.1371/journal.pone.0188842

**Published:** 2017-12-04

**Authors:** Dorota Jędroszka, Magdalena Orzechowska, Raneem Hamouz, Karolina Górniak, Andrzej K. Bednarek

**Affiliations:** 1 Department of Molecular Carcinogenesis, Medical University of Lodz, Lodz, Poland; 2 Department of Pathology, Medical University of Lodz, Lodz, Poland; University of Alabama at Birmingham, UNITED STATES

## Abstract

**Introduction:**

Prostate carcinoma (PRAD) is one of the most frequently diagnosed malignancies amongst men worldwide. It is well-known that androgen receptor (AR) plays a pivotal role in a vast majority of prostate tumors. However, recent evidence emerged stating that estrogen receptors (ERs) may also contribute to prostate tumor development. Moreover, progression and aggressiveness of prostate cancer may be associated with differential expression genes of epithelial-to-mesenchymal transition (EMT). Therefore we aimed to assess the significance of receptors status as well as EMT marker genes expression among PRAD patients in accordance to their age and Gleason score.

**Materials and methods:**

We analyzed TCGA gene expression profiles of 497 prostate tumor samples according to 43 genes involved in EMT and 3 hormone receptor genes (AR, ESR1, ESR2) as well as clinical characteristic of cancer patients. Then patients were divided into four groups according to their age and 5 groups according to Gleason score. Next, we evaluated PRAD samples according to relationship between the set of variables in different combinations and compared differential expression in subsequent groups of patients. The analysis was applied using R packages: FactoMineR, gplots, RColorBrewer and NMF.

**Results:**

MFA analysis resulted in distinct grouping of PRAD patients into four age categories according to expression level of AR, ESR1 and ESR2 with the most distinct group of age less than 50 years old. Further investigations indicated opposite expression profiles of EMT markers between different age groups as well as strong association of EMT gene expression with Gleason score. We found that depending on age of prostate cancer patients and Gleason score EMT genes with distinctly altered expression are: KRT18, KRT19, MUC1 and COL4A1, CTNNB1, SNAI2, ZEB1 and MMP3.

**Conclusions:**

Our major observation is that prostate cancer from patients under 50 years old compared to older ones has entirely different EMT gene expression profiles showing potentially more aggressive invasive phenotype, despite Gleason score classification.

## Introduction

Prostate adenocarcinoma (PRAD) is second the most common solid neoplasm worldwide (incidence rate: 168.3 cases per 100,000 men) [1]. However, there are significant differences in occurrence between regions and country development (70% of accounted PRAD cases are in developed countries). Moreover, increasing age, ethnicity and a family history have been recognized as essential risk factors for PRAD, nevertheless their in-depth significance remains unclear [2].

The current PRAD grading system based on histological expansion rate was developed between 1966 and 1974 by Donald Gleason et al. [3]. Despite scale modifications in 2005 [4] and more recently in 2014 [5] that have dramatically changed original classification, the grading system stays problematic and unclear. In 2013 a new grading system was proposed to minimize overtreatment of low grade prostate tumors detected by PSA tests [6]. Nevertheless, modified Gleason scale remains the golden standard in grading prostate neoplasms. Briefly, according to the guidelines, Gleason score for two the most common patterns is assigned based upon the microscopic appearance of prostate biopsy samples. The more anaplastic and poorly differentiated cells, the higher the Gleason score given, representing a more aggressive character of the tumor. Subsequently, the scores for both patterns are summed to obtain eventual Gleason score ranging from 2–10 (well-differentiated to least differentiated) thus representing biologically similar groups of low, intermediate and high-grades [7].

Epithelial-to-mesenchymal transition (EMT) is a basic mechanism that plays a central role in development, tissue regeneration, architecture and remodeling in physiology as well as pathological migratory properties of cancer cells. In greater detail, EMT describes a process of loss of epithelial character towards gain of mesenchymal properties. This is done mainly due to loss of cell-cell communication, adhesion and reorganization of cytoskeleton leading to a switch from apical-basal to front-rear polarity of cells. Therefore, the EMT may be considered as molecular mechanism of acquisition of invasive properties as well as enhancing tumor aggressiveness and migratory potential through involvement of regulatory pathways (mainly transforming growth factor β (TGF-β), phospahtydylinositol-3-kinase (PI3K) and mitogen-associated protein kinase (MAPK)) and particular genes (E-cadherin, β-catenin, fibronectin, vimentin and matrix metalloproteases) [8]. In fact, since Gleason score quasi represents the tumor aggressiveness it becomes apparent that Gleason may be considered as morphological evidence of EMT and has been hereby described [9]. Many recent studies have focused on *in vivo* significance of EMT with respect to clinical course of the disease, however there are still some discrepancies, which should be investigated [10].

The association between patient age and aggressiveness (and hence Gleason score) of prostate tumor has not been well investigated so far. There are many reports suggesting that younger men experience PRAD of completely different biology compared to those diagnosed at higher age [11]. This raises several important questions how to distinguish aggressive cases from indolent form and regarding the management adjusted to the differential biology of the tumor.

Signaling via androgen receptor (AR) plays a pivotal role in both, the development and function of normal prostate gland as well as tumorigenesis of the prostate. Canonical signaling through AR comprises modulation of transcriptional activity of particular genes through AR nuclear translocation and binding to androgen response elements (AREs) on its targets followed by recruitment or crosstalk with various transcription factors (Tfs)[12]. Surprisingly, recent works have started to prove that not only AR has major influence on predisposing and a PRAD incident, but so do estrogen receptors. Especially, the role of ESR2 is believed to be important in prostate cancer progression. Nevertheless, their significance in PRAD biology and in accordance to patient age remains inconclusive [13].

To date, several studies indicated that sex hormones such as testosterone and estradiol are declining with age in males. More particularly, androgen levels and estrogen levels are unbalanced in men of higher age, it has been showed that androgen loses its significance in favor of estrogen followed by altered body composition and increased BMI in olders [14].

Considering the fact that the levels of androgens and estrogens in men change with age and taking into account the impact of estrogen and androgen hormones in PRAD progression and development, we have studied biological differences of PRAD between younger and older patients. Moreover, we also examined the significance of EMT and signaling via AR and both ERs status in prostate according to patient age and Gleason score.

## Results

### The significance of hormone receptors changes with patient’s age

Firstly, we examined the significance of three hormone receptors: *AR*, *ESR1*, *ESR2* in the biology of prostate cancer regarding the effect of their expression on tumor recurrence according to patient’s age (four age groups: ≤50, 51–60, 61–70, 70> years old) through disease-free survival (DFS) analysis. We found a shift in the significance of the receptors for tumor recurrence; higher rates of *AR* expression are unfavorable for prostate tumor in younger patients, however it loses its significance in the oldest group, in contrast to *ESR1*, where higher expression is generally unfavorable. In greater detail, we identified lowered expression of *AR* as favorable in groups of ≤50 and 51–60 years old (HR>100, p = 0.036; HR = 3.12, p = 0.0027, respectively), whereas in groups of older patients (61–70 and 70> years old) *AR* lost its significance. In contrast, *ESR1* did not show any importance in groups of ≤50 and 70> years old in tumor recurrence, although its lowered expression was associated with better prognosis in remaining groups of 51–60 and 61–70 years old (HR = 7.95, p<0.001; HR = 2.75, p = 0.0053). Finally, lowered expression of *ESR2* correlated with good prognosis in group of 51–60 years old (HR = 2.36, p = 0.033), whereas relatively higher expression was favorable in group of 61–70 years old (HR = 0.31, p = 0.024) while not relevant in the youngest and oldest patients. Summarized results are presented in Table 1. Full results of DFS analysis with Kaplan-Meier plots are enclosed as S1 Fig.

**Table 1:** DFS analysis shows differences in tumor biology according to patient age.

	Age group											
	≤50			51–60			61–70			70>		
Gene	HR^1^	Number of patients in group		HR^1^	Number of patients in group		HR^1^	Number of patients in group		HR^1^	Number of patients in group	
		Low expression^2^	High expression^2^		Low expression^2^	High expression^2^		Low expression^2^	High expression^2^		Low expression^2^	High expression^2^
*AR*	>100*	**12**	**23**	3.12*	**95**	**93**	1.77	136	101	0.28	17	20
*ESR1*	0.24	11	24	7.95**	**176**	**12**	2.75*	**213**	**24**	2.12	12	25
*ESR2*	>100	11	24	2.36*	**165**	**23**	0.31*	**17**	**220**	0.15	21	16

^1^ Hazard ratio.

^2^ “Low expression” is defined as expression values below the cutoff and “high expression” as expression values above the cutoff.

* p-value < 0.05,

** p-value < 0.001

Subsequently, we focused on biological differences between prostate tumors regarding sets of variables such as patient age, Gleason score as well as hormone receptors expression. Patients were dimensionally partitioned by applying the Multiple Factor Analysis (MFA).

**Fig 1:**
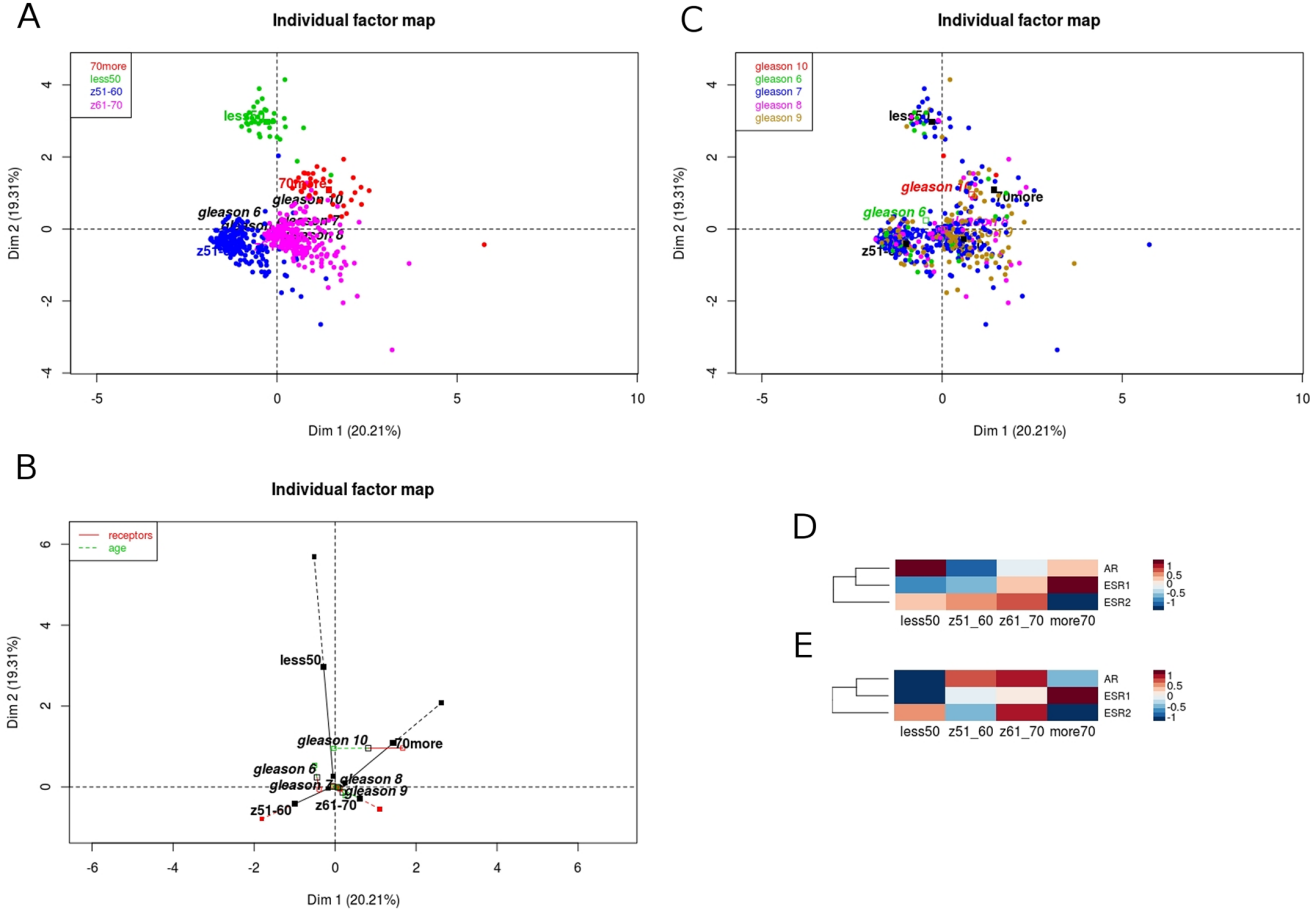
Characteristics of PRAD patients are related to *AR*, *ESR1* and *ESR2* expression, and patient’s age. The expression of *AR*, *ESR1* and *ESR2* indicates partition of PRAD cases into patients age (A), however no associations in Gleason score (B). The projections along Dim2 show distinct grouping of patients aged ≤50 years old according to hormone receptors expression and shift of partitioning of patients of 70> years old along Dim1 (C). Differential expression of particular receptors: shift from high expression of *A*R in the youngest patients towards high expression of *ESR1* in the oldest within tumor samples (D) and lower *AR* expression, but no differences in the expression of both estrogen receptors in matched, adjacent normal samples (E).

First analytical variant comprised combined expression of *AR*, *ESR1* and *ESR2*, patient age and Gleason score as supplementary variable. The analysis resulted in plots of individuals (patients) shown as Fig 1 with 39.52% of the total variability for the first two dimensions. We observed clearly distinct grouping of patients aged ≤50 and 70> years old and more common character of tumors in patients aged 51–70 years old. More specifically, the contribution of all three hormone receptors are pretty similar in the prostate tumorigenesis, however the shift in the significance from androgen in the youngest towards estrogen alpha in the oldest group may be observed in the projections along Dim1 and Dim2 (Fig 1C), while the distribution of Gleason score in the groups remains equable (Fig 1A and 1B). Additionally, as we pointed out higher expression of *ESR1* was unfavorable for tumor recurrence, thus it may indicate that the shift from a high *AR* expression in patients aged ≤50 years old towards high expression of *ESR1* in patients aged 70> years old is caused by the occurrence of andropause in the oldest group (Fig 1D). This finding has been referred to expression rates of hormone receptors in matched, normal adjacent samples. Admittedly, the group sizes were much smaller (≤50: 5, 51–60: 18, 61–70: 25, 70>: 4 normal samples vs ≤50: 35, 51–60: 188, 61–70: 237, 70>: 37 tumor samples), but we found significant changes in expression of hormone receptors: lower expression of *AR* in groups ≤50 and 70> years old and its higher expression in groups 51–70, although no visible changes in both estrogen receptors, possibly indicating that tumors in older patients result from hyperplasia of the prostate and seem to be more normal-like (Fig 1E).

### Prostate tumor aggressiveness is reflected by EMT profile due to different mechanism of transition in younger males

Next, as we identified different profiles of hormone receptors in the youngest vs the oldest patients we investigated further biological effects possibly explaining the aggressive course of the disease in men aged ≤50 years in contrast to the oldest males. Therefore we focused on the mechanism of EMT (which is directly associated with cancer aggressiveness and thus indirectly with Gleason score) through the insight into expression of EMT marker genes in correlation with patients age and Gleason score. Primarily, we selected from the literature 43 well-known EMT markers (Table 2). Subsequently, we applied MFA according to expression of all 43 EMT markers, age groups with Gleason score (supplementary variable) and found distinct profiles of EMT genes in patients aged ≤50 years associated with different response to hormone signaling, with no visible patterns of Gleason distribution (S2A and S2B Fig). In addition, hierarchical clustering of EMT markers according to patients age revealed apparently different character of the tumors diagnosed at age ≤50 years with overexpression of *CTNNB1*, *SMAD2*, *SMAD3*, *TCF4* and *ZEB1* indicating more aggressive clinical course of the tumor due to predominance of mesenchymal pattern in the youngest males (S2D Fig).

**Table 2:** List of genes involved in epithelial-to-mesenchymal transition (based on literature review).

Gene symbol	Gene name	Marker of	References
*CDH1*	Cadherin 1, E-cadherin	epithelial state	[15–17]
*COL4A1*	Collagen type IV alpha 1 chain		
*DSP*	Desmoplakin		
*KRT18*	Keratin 18		
*KRT19*	Keratin 19		
*KRT5*	Keratin 5		
*LAMA1*	Laminin subunit alpha 1		
*LAMA2*	Laminin subunit alpha 2		
*LAMA3*	Laminin subunit alpha 3		
*LAMA4*	Laminin subunit alpha 4		
*LAMA5*	Laminin subunit alpha 5		
*MUC1*	Mucin 1, cell surface assoc.		
*NID1*	Nidogen 1		
*OCLN*	Occludin		
*TJP1*	Tight junction protein 1		
*ACTA2*	Actin 2, alpha 2	mesenchymal state	
*CDH11*	Cadherin 11		
*CDH2*	Cadherin 2, N-cadherin		
*CTNNB1*	Catenin beta 1		
*DDR2*	Discoidin domain receptor tyrosine kinase 2		
*FN1*	Fibronectin 1		
*FOXC2*	Forkhead box C2		
*GSC*	Goosecoid homeobox		
*ITGA5*	Integrin subunit alpha 5		
*ITGB6*	Integrin subunit beta 6		
*KRT8*	Keratin 8		
*LEF1*	Lymphoid enhancer binding factor 1		
*MMP2*	Matrix metallopeptidase 2		
*MMP3*	Matrix metallopeptidase 3		
*MMP9*	Matrix metallopeptidase 9		
*S100A4*	S100 calcium binding protein A4		
*SDC1*	Syndecan 1		
*SERPINH1*	Serpin family H member 1		
*SMAD2*	SMAD family member 2		
*SMAD3*	SMAD family member 3		
*SNAI1*	Snail family transcriptional repressor 1		
*SNAI2*	Snail family transcriptional repressor 2		
*TCF3*	Transcription factor 3		
*TCF4*	Transcription factor 4		
*TWIST1*	Twist family bHLH transcription factor 1		
*VIM*	vimentin		
*ZEB1*	Zinc finger E-box binding homeobox 1		
*ZEB2*	Zinc finger E-box binding homeobox 2		

**Fig 2:**
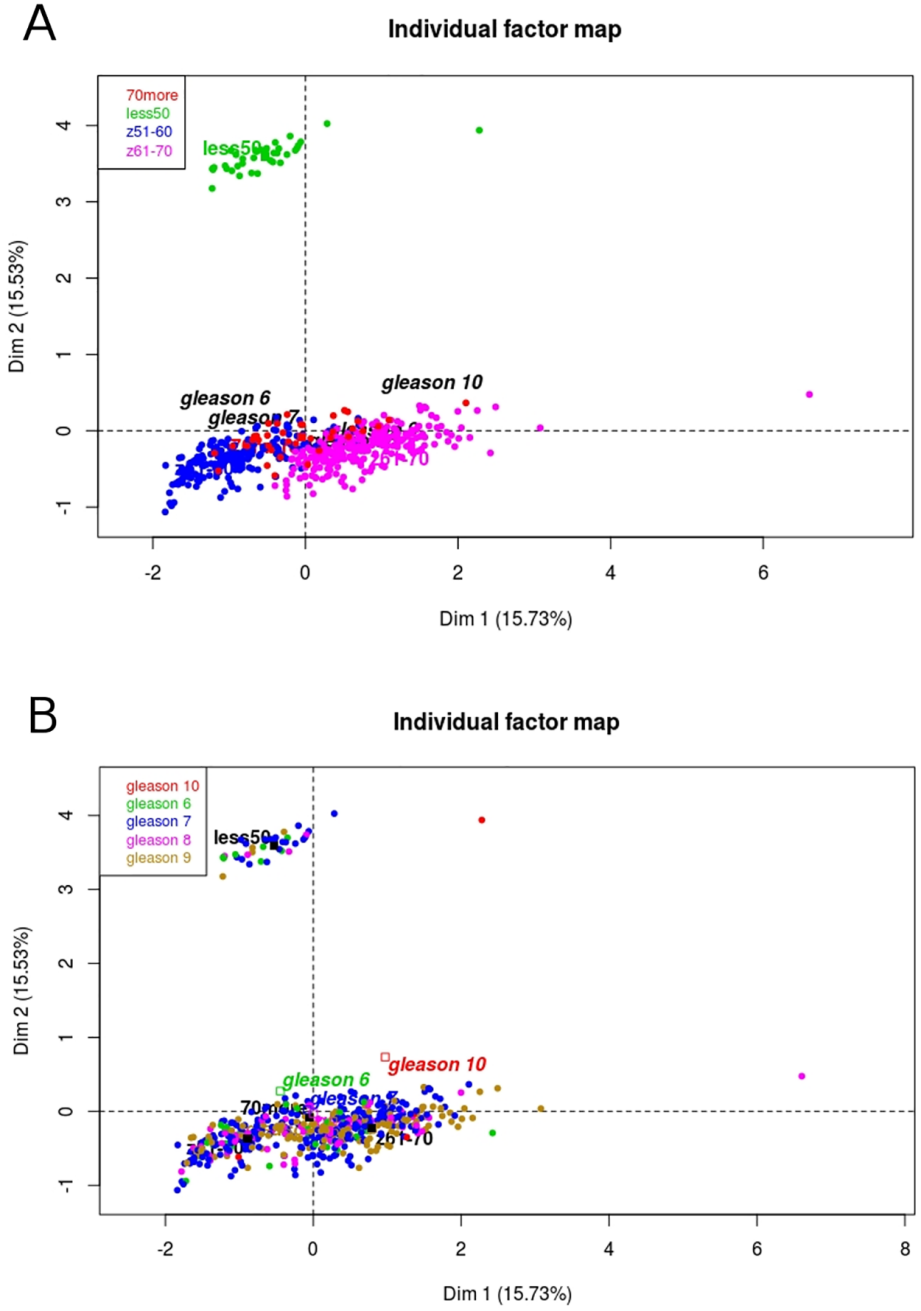
Distinct biology of PRAD patients aged ≤50 years old relates to different profile of EMT. The expression of selected EMT markers indicates completely different partitioning of PRAD patients aged ≤50 years old (A), however equable distribution of Gleason score amongst patients (B). The projections along Dim2 show distinct grouping of patients aged ≤50 years old in association with different profile of selected EMT markers with simultaneous similar character of tumor diagnosed in older males (C).

**Fig 3:**
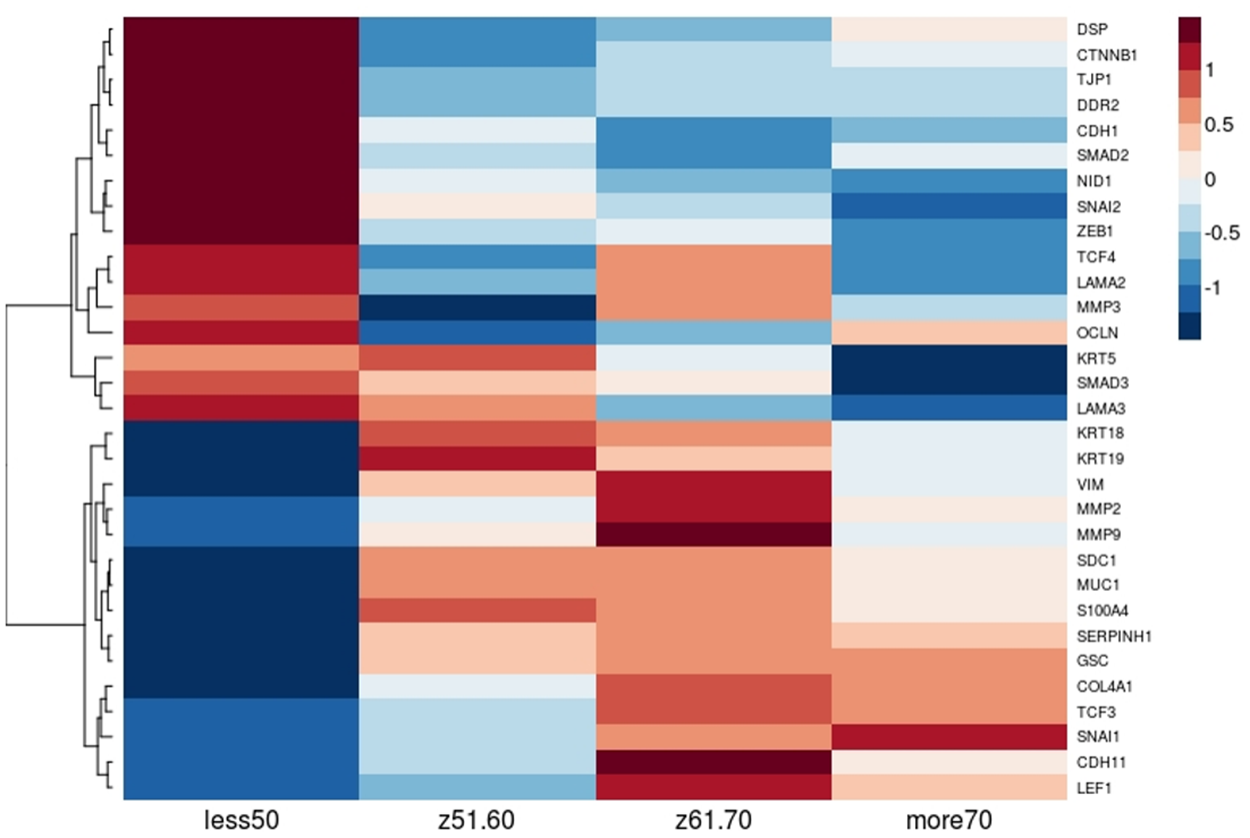
Characteristics of PRAD patients are related to different EMT profile in association with patient’s age. The opposite profiles of expression of particular EMT markers in the youngest vs the oldest patients have been identified: heightened expression of *CDH1* and *CTNNB1* in patients below 50 years old and elevated expression of *SNAI1* and simultaneous lowered expression of *CDH1* and *CTNBB1* in older patients.

Furthermore, from the initial 43 EMT markers we subsequently focused on the 31 genes (hereinafter called as selected), which were mostly differentiating patients ≤50 vs 70> years old. As can be seen in the Fig 2, by applying MFA the youngest patients were even more distinct with 31.26% of the total variability, while the distribution of Gleason score remained equable. The heatmap representing contrasting profiles of selected EMT markers in patients according to age group may be seen in the Fig 3.

**Fig 4:**
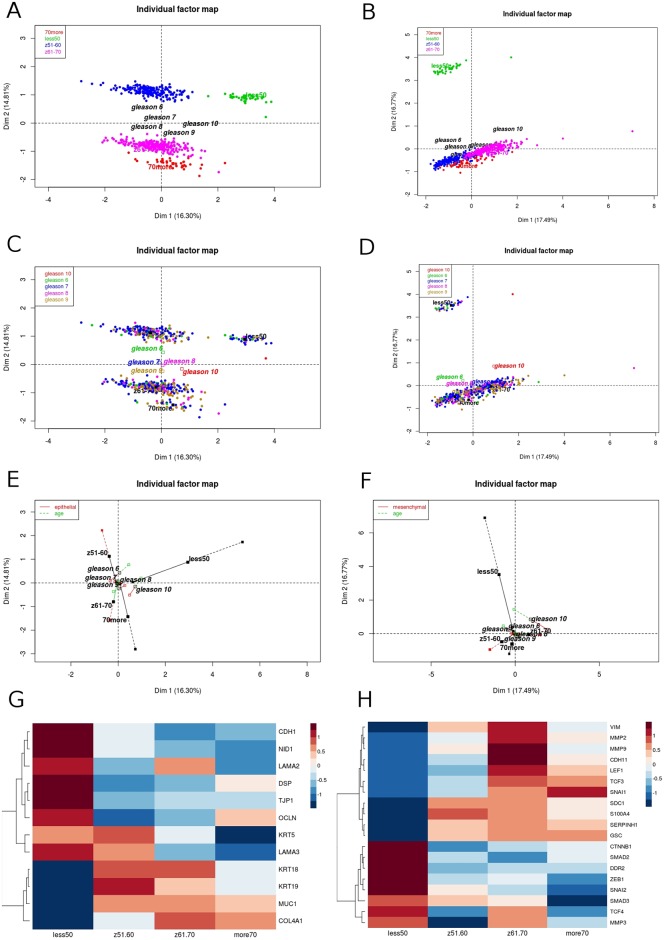
Prostate tumors diagnosed at age ≤50 and 70> years old show shift in characteristics regarding expression of the mostly differentiating epithelial vs mesenchymal state markers. The expression of epithelial (A, C) vs mesenchymal (B, D) state markers in separate partitions PRAD patients oppositely. The projections representing contribution of epithelial (E) and mesenchymal (F) state markers along the dimensions indicate significant shift in the EMT model in the age groups, especially in the youngest and the oldest males. In addition, the shift towards more aggressive mesenchymal character of the tumor is observable in the contrasting expression of particular genes involved in the EMT (G) epithelial state markers, H) mesenchymal state markers).

Subsequently we evaluated epithelial apart from mesenchymal state markers and their separate contribution to biology of the tumors in respect to patients age as well as dimensional partitioning of PRAD cases. The variant considering all divided EMT markers enabled us to identify two phenomena: first, reversed profiles between epithelial and mesenchymal states especially in groups of ≤50 and 70> years old (S3A and S3B Fig) and second, significant shift in projections along Dim2 between the youngest and the oldest groups of patients (S3C and S3D Fig). Therefore, differential expression of EMT genes shows shift towards more mesenchymal and thus more aggressive phenotype of tumors in patients ≤50 years old (S3E and S3F Fig). Similar partitioning was found in markers after selection of the most differentiating genes with slight shift in epithelial markers regarding group aged ≤50 years old (Fig 4).

### Profile of EMT markers reflects Gleason score

**Fig 5:**
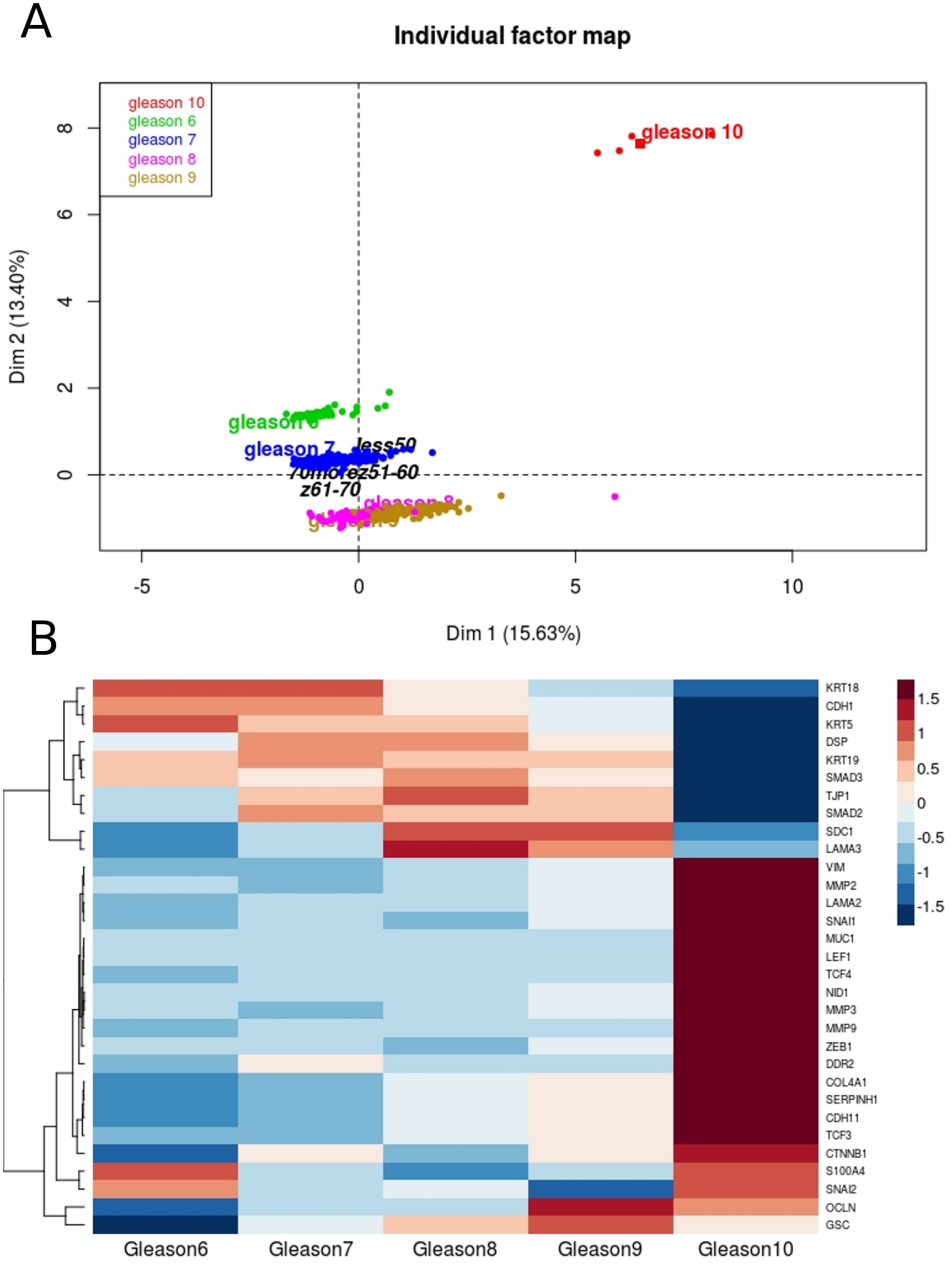
Expression of EMT markers differentiates well Gleason score. A) Combined expression of selected EMT markers partitions Gleason score along the PCA dimensions. B) Expression of particular EMT markers changes with Gleason score.

Furthermore, as EMT signature has been found significantly contrasting PRAD patients according their age and tumor biology, we evaluated the association of Gleason score and EMT markers (which *a priori* should be true). As expected, we found that expression of selected EMT markers pretty well differentiates Gleason scores into separate partitions along with the dimensions with 29.03% of total variability, thus bearing out the strong relationship between tumor aggressiveness characterized on the one hand by expression of EMT markers and on the other, by clinical parameter—Gleason score (Fig 5A). Noteworthy, as shown in the Fig 5B, the expression of particular genes involved in EMT changes along with higher Gleason scores, hence represents increase in aggressiveness of prostate tumor.

### Validation test

We performed validation of hierarchical clustering analysis regarding expression of EMT marker genes in association with Gleason score. Our results validation was performed on mRNA expression data from Prostate Adenocarcinoma (MSKCC, Cancer Cell 2010) cohort from cBioportal due to the sufficient number of patients and available information of Gleason score. Those gene expression data comes from different cohort of patients and were obtained using microarray analysis. Although patients with Gleason 10 are missing, this analysis confirmed general tendency that profiles of EMT markers are distinct among Gleason score especially in patient with high Gleason score (S4 Fig). Several mesenchymal markers like *SNAI1* and its target genes *MMP3* and *MMP9* are elevated while crucial epithelial markers *CDH1*, *KRT5* and *KRT19* are significantly decreased.

### Common Gleason score, but different molecular profiles of EMT markers according to age group

**Fig 6:**
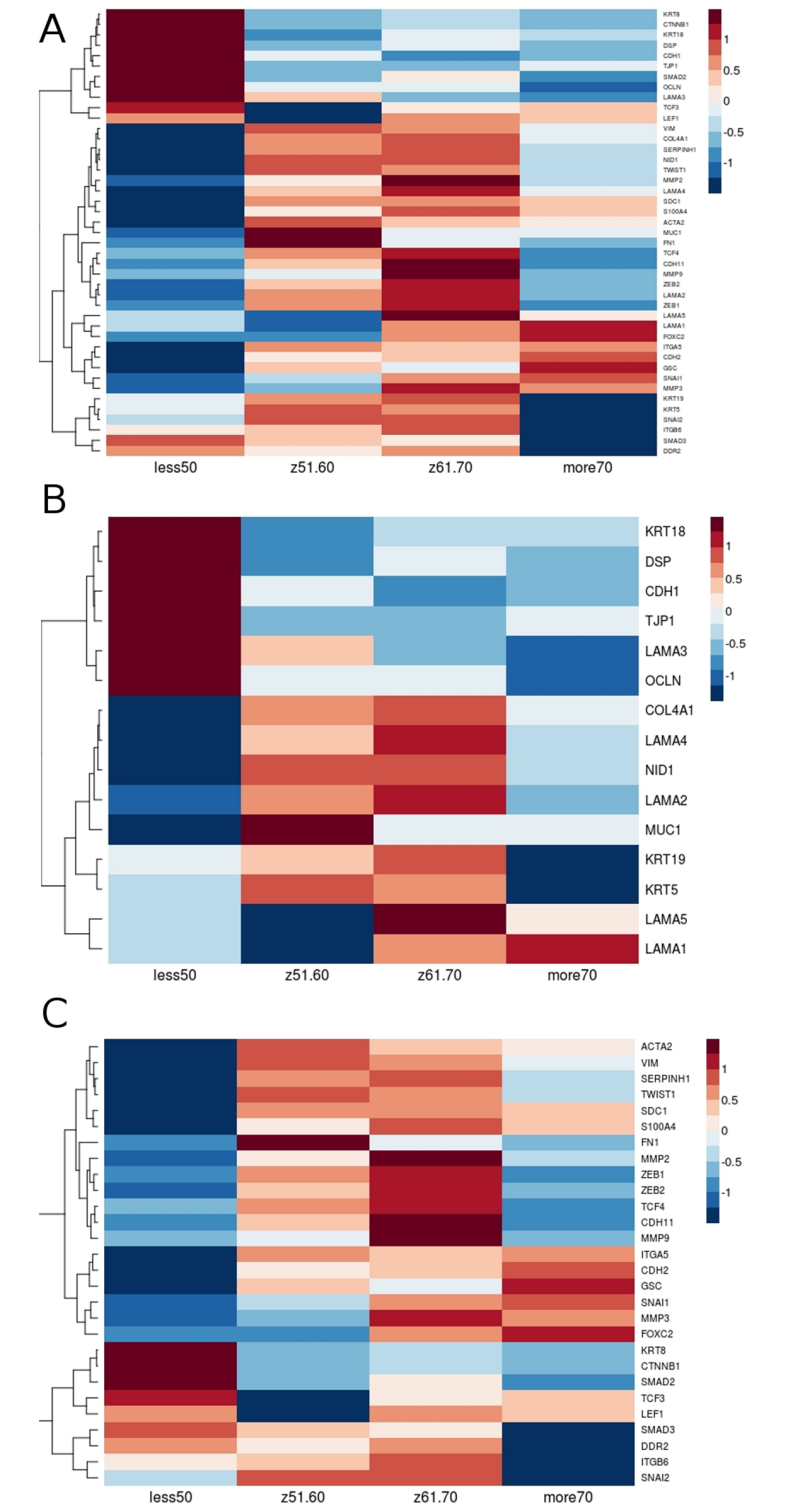
The same Gleason score differs in age groups in expression of EMT markers. The profiles of EMT markers are distinct between age groups within the same Gleason score (A). Differential expression of epithelial (B) vs mesenchymal (C) markers in age groups within the same Gleason score.

We demonstrate the expression profiles of all EMT markers in higher Gleason score groups (the most aggressive cases: 8+9 with exception of Gleason 10 due to insufficient number of patients) according to patients age. We observed that the same Gleason score differs in the EMT signature according to patient age. In particular, we found primarily overexpression of *CTNNB1*, *CDH1*, *SMAD2*, *SMAD3*, *TCF3*, *LEF1* in younger patients and overexpression of *SNAI1* and underexpression of *KRT5*, *KRT19*, *OCLN*, *CDH2* and *MUC1* which clearly indicates different characteristics of prostate tumor in younger vs older patients (Fig 6).

### Mutations play marginal role in prostate tumorigenesis

Cancer has been recently considered as genetic disorder, which results from many alterations at molecular level with mutations among others. To complete the insight into prostate tumorigenesis biology we additionally examined the significance of the mutations borne by patients, although their role seems marginal (does not exceed 20% of all cases). Tables 3 and 4 present mutational reports from COSMIC database vs mutations identified within PRAD cohort. Most mutations in PRAD affect *TP53* gene, as we should suspect. Second in mutation rate is androgen receptor, the castrate resistant cancer, thought there is less than 10% mutations found in COSMIC reported prostate cancer. Therefore, as in the most sporadic cancers there is no specific highly represented mutations in PRAD. Nevertheless, in the era of personalized medicine, even relatively rare mutations can be important in treatment profiling.

**Table 3:** Top 20 mutations reported by COSMIC database vs PRAD cohort.

	Mutations			
	COSMIC		TCGA	
Gene symbol	no.	%	no.	%
*TP53*	2506	15	497	12.2
*AR*	2992	9		0.6
*SPOP*	1802	8		11.4
*PTEN*	2339	7		3.4
*KMT2C*	1436	5		6
*KMT2D*	1438	5		5.8
*FOXA1*	1670	5		5.6
*FAT4*	1436	5		2.4
*LRP1B*	1436	4		4.8
*KRAS*	2831	3		0.4
*ATM*	1503	3		4.4
*ZFHX3*	1436	3		3.2
*CTNNB1*	1958	3		2.6
*APC*	1676	3		2
*EGFR*	2083	2		0.6
*PIK3CA*	1944	2		2.8
*SPEN*	1462	2		1.4
*FAT1*	1436	2		0.8
*GRIN2A*	1444	2		2.2
*BRCA2*	1603	2		1.8

**Table 4:** Top 20 mutations identified within PRAD cohort.

	TCGA	
Gene symbol	no.	%
*FRG1BP*	97	19.4
*TP53*	61	12.2
*SPOP*	57	11.4
*MUC17*	33	6.6
*KMT2C*	30	6
*KMT2D*	29	5.8
*FOXA1*	28	5.6
*NBPF1*	27	5.4
*SYNE1*	27	5.4
*SPTA1*	26	5.2
*LRP1B*	24	4.8
*KRTAP4-11*	24	4.8
*NBPF10*	23	4.6
*ATM*	22	4.4
*ZAN*	22	4.4
*CHEK2*	21	4.2
*FAT3*	19	3.8
*OBSCN*	18	3.6
*RGPD8*	18	3.6
*PTEN*	17	3.4

## Discussion

### Role of androgen and estrogen receptors

In our study we aimed to evaluate DFS prognostic effect of androgen and estrogen receptors gene expression according to patient age in prostate cancer. Multifaceted association of receptors gene expression with DFS inclined us to explore biological and clinical differences between age groups of patients with prostate adenocarcinoma. For this purpose we analyzed epithelial to mesenchymal transition marker genes which are responsible for cancer cell aggressiveness, metastasis and poor prognosis in various tumors. We performed multiple factor analysis to integrate the relationship between the groups of variables (age, Gleason score and genes expression) describing the individuals (patients). Our results showed that accordingly to patient age, gene expression and Gleason score, the most diverse group form patients below 50 years old.

Androgen signaling is the key regulator in normal development and maintenance of the prostate growth and function. There is a growing body of evidence supporting involvement of the androgen receptor in the progression of hormone-sensitive but also hormone-insensitive prostate cancer. Moreover, the majority of prostate cancer both at primary and metastatic sites are characterized by AR presence regardless of stage and grade. Zegarra-Moro et al. demonstrated that *AR* is critical for development and proliferation of androgen-refractory prostate tumor cell lines LNCaP-Rf and LNCaP-C4 [18]. *AR* overexpression and amplification in prostate cancer cells has been shown to correlate with lower recurrence-free survival rates [19] as well as the transition from hormone sensitive to resistant prostate tumor [20,21]. Patients with high levels of *AR* in malignant epithelial cells and a reduction of *AR* positive nuclear in peritumoral stromal cells had increased risk of relapse following radical prostatectomy [22]. Consistent with the earlier reports, our analysis has shown that *AR* decreased expression is significantly associated with better DFS prognosis but only for relatively young patients below 50 years old and for the age group 51–60. Moreover, for patients under 50 years old, *AR* expression profile points to unfavorable prognosis due to the fact that in this group *AR* expression is upregulated in comparison to normal adjacent tissue (Fig 1D and 1E). This could mean that through the high AR level and different expression profile of *AR* compared to favorable prognosis, younger patients have even more aggressive type of cancer than patients in more advanced age.

In addition to role of androgens in prostate carcinogenesis, estrogen and thus estrogen receptors play an important role in molecular mechanism of tumorigenesis through proliferation, apoptosis, invasiveness or epithelial-to-mesenchymal transition (EMT)[23–26]. In prostate ERα is mainly expressed in stromal cells, whereas ERβ is localized mainly in epithelial cells [27,28]. Studies have shown the oncogenic role of *ERα* in various prostate cancer cell lines including PacMetUT1, C4-2, 22Rv1 and LNCaP [27] but also in patients with aggressive high Gleason score tumors [28]. Moreover, *ERα* has been found to mediate bone and lung metastasis and induce EMT program in cancer cells [27]. Gene polymorphisms in the *ERα* and *ERβ* locus have been shown to be significantly associated with prostate cancer risk, overall or by grade, and stage [29]. Studies on animal model (ER-knockout, ERKO mice) show that ERβ-knockout mice develop prostate cancer after stimulation of testosterone or other sex hormones, whereas ERα KO do not [30]. Furthermore, Leav et al. demonstrated diminished expression of *ERβ* during prostatic carcinogenesis and tumor progression [31]. Our analysis showed, for the first time, that for patients older than 50 years old, lowered expression of *ERα* in prostate cancer cells indicates better prognosis, which would coincide with its oncogenic character. On the other hand, we observed an association of favorable DFS prognosis with *ERβ* decreased expression in younger group of patients, whereas in patients older than 60 years old decreased *ERβ* expression indicates bad prognosis. Together our data would seem to suggest that *ERα* may play an oncogenic role for patient age above 50. In this context it is worth to mention that estrogen receptors may have significant influence on prostate cancer aggressiveness especially in patients older than 70 years old. Decreased level of *ERα* and increased level of *ERβ* are correlated with better prognosis for older patients while in younger groups of patients better DFS prognosis is associated with opposite expression profiles of hormone receptor genes (S1 Fig). Moreover the attention should be paid to fact that according to MFA analysis, gene expression of sex hormone receptors is very similar during disease progression (Fig 1C), however there is a visible change in gene expression dominance from *AR* in younger patients to *ESR1* and *ESR2* in older patients (Fig 1D).

We also investigated the mechanism of epithelial-to-mesenchymal transition and have looked into differences in the expression of EMT genes according to patient age and Gleason score. Since it is well known that Gleason score is a clinical representation of aggressiveness, it should be a direct association between Gleason grade and EM transition. And it is in fact, that that Gleason grades are clearly separated from the other according to the EMT genes, with Gleason 10 as the most distant phenotype (individual factor map, Fig 5A). Moreover, the expression of specific EMT genes change along with higher Gleason score (Fig 5B). Finding that there is a switch between the androgen and estrogen receptors gene expression depending on the patient age, as well as Gleason score association with EMT we assumed that there may be some distinct differences between younger and older patients in the profile of epithelial to mesenchymal transition mechanism in prostate cancer progression.

### Epithelial-to-mesenchymal transition in prostate cancer

Epithelial-to-mesenchymal transition is a biological process in which loss of epithelial cell phenotype leads to destabilization of intercellular interaction and cell polarity. Therefore inducing tissue disintegration. One of the major changes is loss of E-cadherin in cellular membrane. At the same time due to gain of expression of genes like vimentin and N-cadherin, cells acquire mesenchymal phenotype facilitating migration, invasion and survival in an anchorage-independent environment. EMT is observed under physiological condition because it underlies many phases of embryonic development. However it may occur in many pathological states such as tumorigenesis and metastasis [15,32,33]. Its relevance in cancer progression has been demonstrated in the context of direct repression of E-cadherin promotor by group of transcription factor from *SNAIL*, *TWIST* and *ZEB* families [34,35]. E-cadherin plays an important role in maintenance the epithelial integrity [36] and its decreasing is well described in many cancers. In prostate cancer aberrant expression of E-cadherin is characteristic for high-grade tumor [37] and associated with poor overall survival of patients [38]. Our analysis shown that expression of *CDH1* was actually reduced in prostate tumors from patients older than 50 years old (Fig 3). Interestingly, that in group of prostate cancer patients under the age of 50, E-cadherin expression is greatly enhanced in tumors together with the overexpression of β-catenin gene. In normal cells CTNNB1 forms a complex with CDH1 and stabilizes cell to cell adhesion [32,39]. However, as a result of aberrant activation of Wnt/β-catenin pathway in the cancer, cytoplasmic accumulation of β-catenin and proliferation of prostatic cells were observed [40–42]. It may be possible that due to enhanced activity of strongly oncogenic Wnt pathway and the relatively high expression, E-cadherin loses its normal function. Moreover, the oncogenic effect of *CTNNB1* may be further enhanced by the synergistic activity of *SMADs*, *TCF* and *LEF* (Figs 4E and 6C) that was also confirmed in Labbe et al. study that shown that activities of these genes products strongly induce target genes transcription at when all three transcription factors are involved [43]. Another proposed hypothesis says that along with the progress of EMT, the neoplastic cells regain *CDH1* expression in established metastatic foci [44]. Re-acquisition of cell-cell adhesion ability typical for epithelial phenotype could be advantageous for tumor growth and escalated invasiveness. Saha et al. found overexpression of both adhesion-associated proteins in metastatic cancer cells and their reduced expression in primary prostate cancer cells [45]. In contrast, we observed that relatively high *CDH1* expression in primary tumors from younger patients is associated with higher Gleason score characteristic for local invasiveness. Our analysis (Fig 6B and 6C) show that younger patient cancers with Gleason score equal 8 and 9 are characterized by significant overexpression both of *CDH1* and *CTNNB1* (Fig 6B and 6C). On the other hand decreased expression of these two genes are observed for patients above the age of 50 also when Gleason score is high. Furthermore, older patients, especially over the age of 70, are characterized by increased level of *CDH2* expression (Fig 6C). It may reflect a phenomenon called “cadherin switch” during which loss of epithelial E-cadherin is accompanied by production of mesenchymal N-cadherin [46–48]. *CDH2* is typically expressed in mesenchymal cells and its upregulation in cancer promotes cell motility and invasiveness [49]. In prostate cancer, the switch between E-cadherin and N-cadherin was reported for high Gleason grade tumors [50]. This data suggest that according to patient age increased or decreased level of *CDH1* and *CTNNB1* has a significant influence on the aggressiveness of the tumor and is associated with different profiles of epithelial-mesenchymal transitions.

Recently, upregulation of several E-cadherin transcriptional repressors like Snail, Twist and Zeb families was found to be crucial for repressing the E-cadherin and thus redirecting cells toward mesenchymal phenotype [34]. *ZEB1* overexpression is associated with tumor cells migration and invasion and together with inhibition of E-cadherin gene expression mediates progression to metastasis [51]. A recent report demonstrated that high *ZEB1* expression directly correlates with high Gleason grade prostate adenocarcinoma [52,53] that is also demonstrated by our analysis (Fig 5B). Alike *ZEB1* also *SNAI1* and *SNAI2* are strongly associated with high Gleason score 10 (Fig 5B), indicating them as markers of an aggressive and advanced prostate cancer phenotype. On the other hand, we observed *ZEB1* decreased expression in tumors of Gleason score 8 and 9 from patients groups aged under 50 and over 70 years. Using in vitro model Drake et al. established for TEM4-18 prostate cancer cells which underwent EMT, loss of *ZEB1* leads to upregulation of epithelial marker *CDH1*, enabling gain of epithelial character [51]. Together with our analysis this suggests that younger patients have even more advanced subtype of prostate cancer; in terms of aggressiveness due to the enhanced E-cadherin (together with β-catenin) level leading to more metastatic phenotype. On the other hand, for older patients low expression of *ZEB1* could be compensated by other EMT regulator such as *SNAI1* (Fig 6C). For example, several studies demonstrated that *SNAI1* is a master regulator sufficient for induction of EMT [54,55]. Additionally *SNAI1* is crucial for expression regulation of epithelial genes such as *KRT18*, *OCLN* and *MUC1* [56], as well as enhanced expression of mesenchymal markers such as *VIM*, *MMP 2*,*-9* [57]. Our investigation supports this findings due to the fact that along with the elevated level of *SNAI1* there is significant loss of *KRT5*, *KRT19*, *OCLN*, *CDH1*, *MUC1* and several other epithelial marker genes in patients older than 70 years with Gleason score 8 and 9. Similarly, expression of *KRT18*, *CDH1* and *OCLN* are decreased in younger patients (of the age group 61–70 years old) but at the same time much more mesenchymal markers which are targets of *SNAI1* are increased (*MMP2*, *MMP3*, *MMP9*, *ZEB1*, *ZEB2*) (Fig 6B and 6C).

Metalloproteases are important factors involved in degradation of extracellular matrix and basement membrane thus contribute to the alteration of cell-matrix adhesion [58,59]. Compiling all data it is becoming evident that according to age, patients with prostate cancer have various mechanism of epithelial to mesenchymal transition. This agrees with clinical data showing that prostate cancer in younger patients is different than in older in a view of survival rate. Specifically, Lin et al. observed that among all men with high grade tumor or locally advanced cancer at diagnosis, younger males had significantly decreased overall survival and disease specific survival [60]. Moreover, Merrill and Bird found that among males diagnosed with advanced or unknown stage or grade prostate cancer, the youngest (<50 y.o.) and the oldest (>80 y.o.) patients were characterized by the poorest prognosis [61]. There are also some new evidences that males aged under 50 years had similar pathological tumor characteristic, histological grade, disease stage, PSA level and biochemical free survival compared to the older population [62,63]. Our finding shows fundamental molecular differences between prostate cancers form young and old patients. The most distinct group according to switch in epithelial-mesenchymal profile seems to be group of patients younger than 50 years old. In this group crucial transcription regulators *ZEB1* and *SNAI1* as well as many mesenchymal markers like *VIM*, *CDH2*, *MMP2*, *MMP3*, *MMP9* are downregulated. But at the same time these patients are characterized by overexpression of the adhesion proteins *CDH1* and *CTNNB1* which according to the literature could be advantageous for invasiveness and colonization. On the other hand, reversed profiles of epithelial and mesenchymal markers could be seen in older prostate cancer patients. In cancers from patients older than 70 years more significant seems to be the upregulation of key transcription factor *SNAI1* and decreased expression of genes responsible for epithelial phenotype *KRT19*, *CDH1*, *OCLN* and *MUC1*. Summarizing, our analysis shows distinct and biologically significant differences in expression profile of sex hormone receptors as well as epithelial to mesenchymal transition genes that are associated with disease progression depicted as Gleason score. However, despite of Gleason score, distinct profiles of EMT genes expression may explain more aggressive phenotype of prostate cancer in younger patients.

## Materials and methods

We performed The Cancer Genome Atlas (TCGA) RNA-Seq expression profiling (level 3 RNASeqV2, RSEM normalized) and clinical characteristics of 499 PRAD patients (http://cancergenome.nih.gov/, data status of Jan 28, 2016). The methods of biospecimen procurement, RNA isolation and RNA sequencing were previously described by The Cancer Genome Atlas Research Network [64].

Afterwards, we combined RNA-Seq data with patient clinical outcome. Samples with any missing clinical or expression values were excluded from further considerations. Finally, we qualified a total of 497 samples.

To determine the relevance of patient age and its associations with hormone receptors (*AR*, *ESR1*, *ESR2*) regarding recurrence of the disease, we divided patients into four groups in accordance to their age: ≤50 y.o. (35 patients), 51–60 y.o. (188 patients), 61–70 y.o. (237 patients) and 70> y.o. (37 patients). Clinical characteristics of cohort patients are shown in Table 5.

**Table 5:** Clinical characteristics of PRAD cohort with specification by age groups.

	Age group			
	≤50	51–60	61–70	70>
Characteristics	total	total	total	total
Age at diagnosis				
median (range)	48 (41–50)	56 (51–60)	65 (61–70)	72 (70–78)
Pre-operative PSA				
median (range)	6.8 (0.8–107)	7.6 (1.7–96.4)	7.35 (0.7–87)	9.45 (1.7–45)
Gleason score				
2+4	-	-	-	1
3+3	7	23	11	3
3+4	13	63	60	10
4+3	6	33	57	5
8≥	9	69	109	18
Tumor cellularity (pathology)				
<20%	1	6	5	-
21–40%	4	8	12	3
41–60%	9	50	84	10
61–80%	12	88	99	17
81–100%	8	29	31	5
NA	1	7	6	2
PSA recurrence				
yes	3	21	34	-
no	27	141	174	29
NA	5	26	29	8

### Significance of hormone receptors in prostate adenocarcinoma

We examined the significance of hormone receptors (*AR*, *ESR1*, *ESR2*) by applying the DFS analysis. According to the calculated cutoff points we were able to split patients into subgroups of favorable/unfavorable recurrence prognosis with regards to the expression level of particular hormone receptor. The analysis was performed separately for each age subset using freely available Cutoff Finder R script [65]. Clinical characteristics defining DFS were as follow: “patient.days_to_last_followup” for survival time and “patient.follow_ups.follow_up.person_neoplasm_cancer_status” for event. For DFS computation, significance of correlation with survival variable as a method for cutoff point optimization were chosen. Briefly, it is defined as the point with the most significant split. Additionally, hazard ratios (HRs) including 95% confidence intervals (CI) were calculated [65]. Differences in DFS between favorable and unfavorable groups were presented in form of Kaplan—Meier plots with p—values calculated (log—rank test, p<0.05).

### Multiple Factor Analysis (MFA)

We performed dimensional grouping through MFA of PRAD patients according to sets of variables in order to determine the relevance of patients’ age, Gleason score and their association with hormone receptors (*AR*, *ESR1*, *ESR2*), and EMT marker genes. Primarily, 43 genes known as markers of epithelial-to-mesenchymal transition have been selected from the literature (Table 2) [15–17].

The MFA was conducted in four variants according to the following parameters: 1) partitioning regarding the age groups and combined effect of expression of hormone receptors (*AR*, *ESR1*, *ESR2*) as well as Gleason score (supplementary variable); 2) partitioning regarding the age groups and combined effect of 43 EMT marker genes as well as Gleason score (supplementary variable); 3) partitioning regarding the age groups and combined effect of epithelial/mesenchymal state markers; 4) partitioning regarding the Gleason score and EMT marker genes. The MFA was applied using packages: FactoMineR and factoextra [66] within R environment [67].

### Hierarchical clustering

Simultaneously to MFA, we performed hierarchical clustering to examine differences in the expression of particular genes. Clustering was conducted using gplots, NMF and RColorBrewer R packages with pairwise distance measure based on Pearson correlation and complete agglomeration method.

We next cross-validate our findings with alternative prostate cancer study. Due to the lack of RNASeq data resources we chose microarray data from Prostate Adenocarcinoma (MSKCC, Cancer Cell 2010) study which is available in cBioPortal. The clinical parameter not include age information, nevertheless we could perform hierarchical clustering analysis using Gleason score information. To determine the relevance of patients Gleason score and its association with EMT marker genes we divided patients into four groups according to their Gleason score: Gleason 6 (41 patients), Gleason 7 (76 patients), Gleason 8 (11 patients), Gleason 9 (11 patients).

### Summary of mutation significance in prostate cancer

Additionally, we analyzed PRAD patients regarding mutations using cBioPortal (http://www.cbioportal.org/) tools. The results have been validated with COSMIC database (Catalogue of somatic mutations in cancer; http://cancer.sanger.ac.uk/cosmic).

## Supporting information

S1 Fig Kaplan-Meier curves for DFS analysis. Panels A-D show *AR* in groups of age ≤50, 51–60, 61–70, 70> years old, respectively; panels E-H show *ESR1* in groups of age ≤50, 51–60, 61–70, 70> years old, respectively; panels I-L show *ESR2* in groups of age ≤50, 51–60, 61–70, 70> years old, respectively.
(TIFF)

S2 Fig Characteristics of PRAD patients according to expression of EMT markers and age groups. The expression of EMT markers indicates partition of PRAD cases into patients age (A), however no associations in Gleason score (B). The projections along Dim2 show significant contribution of EMT in distinct partitioning of patients of age ≤50 years old (C). The grouping results from opposite profiles of expression of particular EMT markers in the youngest vs the oldest patients: heightened expression of *CDH1*, *CTNNB1*, *MMP3*, *KRT5*, *SMAD3* with simultaneous lowered expression of *KRT18*, *KRT19*, *VIM*, *MMP2*, *COL4A1*, *CDH11*, *CDH2*, *ITGA5* (D).
(TIFF)

S3 Fig Prostate tumors diagnosed at age ≤50 and 70> years old show opposite characteristics regarding expression of epithelial vs mesenchymal state markers. The expression of epithelial (A) vs mesenchymal (B) state markers in separate partitions PRAD patients oppositely. The projections representing contribution of epithelial (C) and mesenchymal (D) state markers along the dimensions indicate significant shit in the EMT model in the age groups, especially in the youngest and the oldest men. In addition, the shift towards more aggressive mesenchymal character of the tumor is observable in the contrasting expression of particular genes involved in the EMT (E) epithelial state markers, F) mesenchymal state markers).
(TIFF)

S4 Fig Expression of particular EMT markers changes with Gleason score in patients from Prostate Adenocarcinoma (MSKCC, Cancer Cell 2010) cohort. (TIFF)
