# Supplementary figures and images for: Markers of epithelial-to-mesenchymal transition reflect tumor biology according to patient age and Gleason score in prostate cancer
Source: PLoS One. 2017 Dec 4;12(12):e0188842. doi: 10.1371/journal.pone.0188842 (PMC5714348; doi:10.1371/journal.pone.0188842)

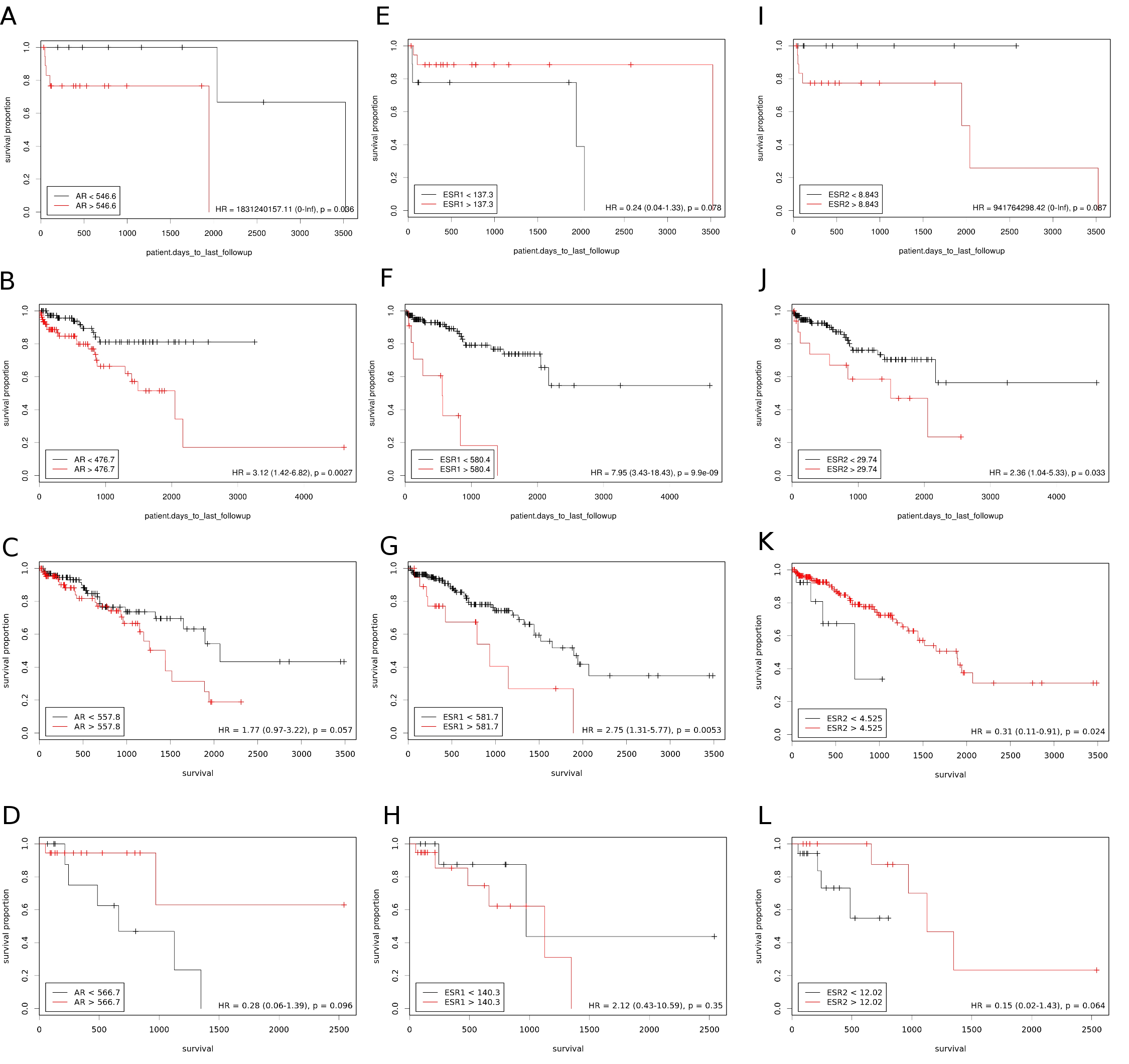

Supplement: S1 Fig — Panels A-D show AR in groups of age ≤50, 51–60, 61–70, 70> years old, respectively; panels E-H show ESR1 in groups of age ≤50, 51–60, 61–70, 70> years old, respectively; panels I-L show ESR2 in groups of age ≤50, 51–60, 61–70, 70> years old, respectively. (TIFF) [file pone.0188842.s001.tiff]

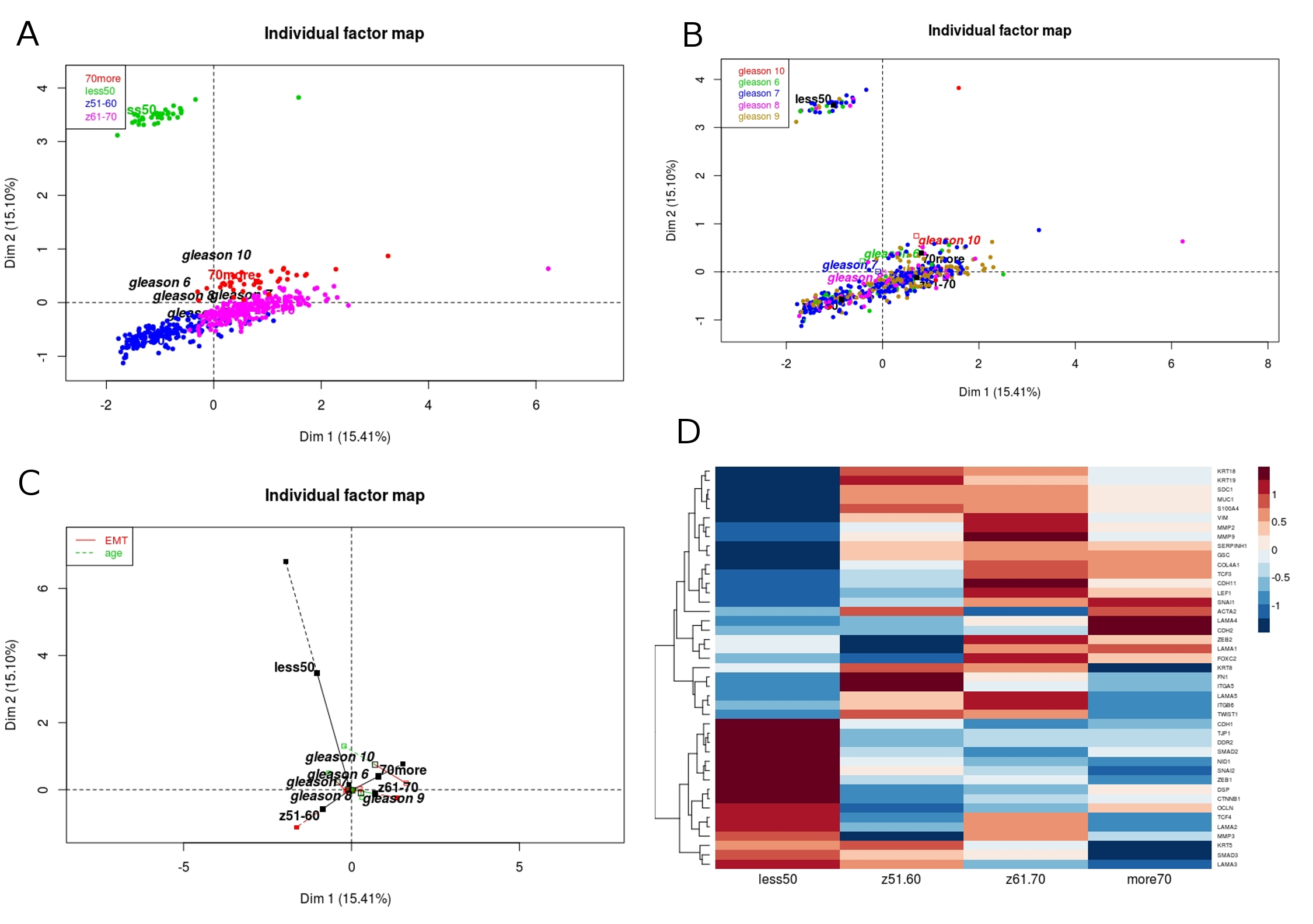

Supplement: S2 Fig — The expression of EMT markers indicates partition of PRAD cases into patients age (A), however no associations in Gleason score (B). The projections along Dim2 show significant contribution of EMT in distinct partitioning of patients of age ≤50 years old (C). The grouping results from opposite profiles of expression of particular EMT markers in the youngest vs the oldest patients: heightened expression of CDH1, CTNNB1, MMP3, KRT5, SMAD3 with simultaneous lowered expression of KRT18, KRT19, VIM, MMP2, COL4A1, CDH11, CDH2, ITGA5 (D). (TIFF) [file pone.0188842.s002.tiff]

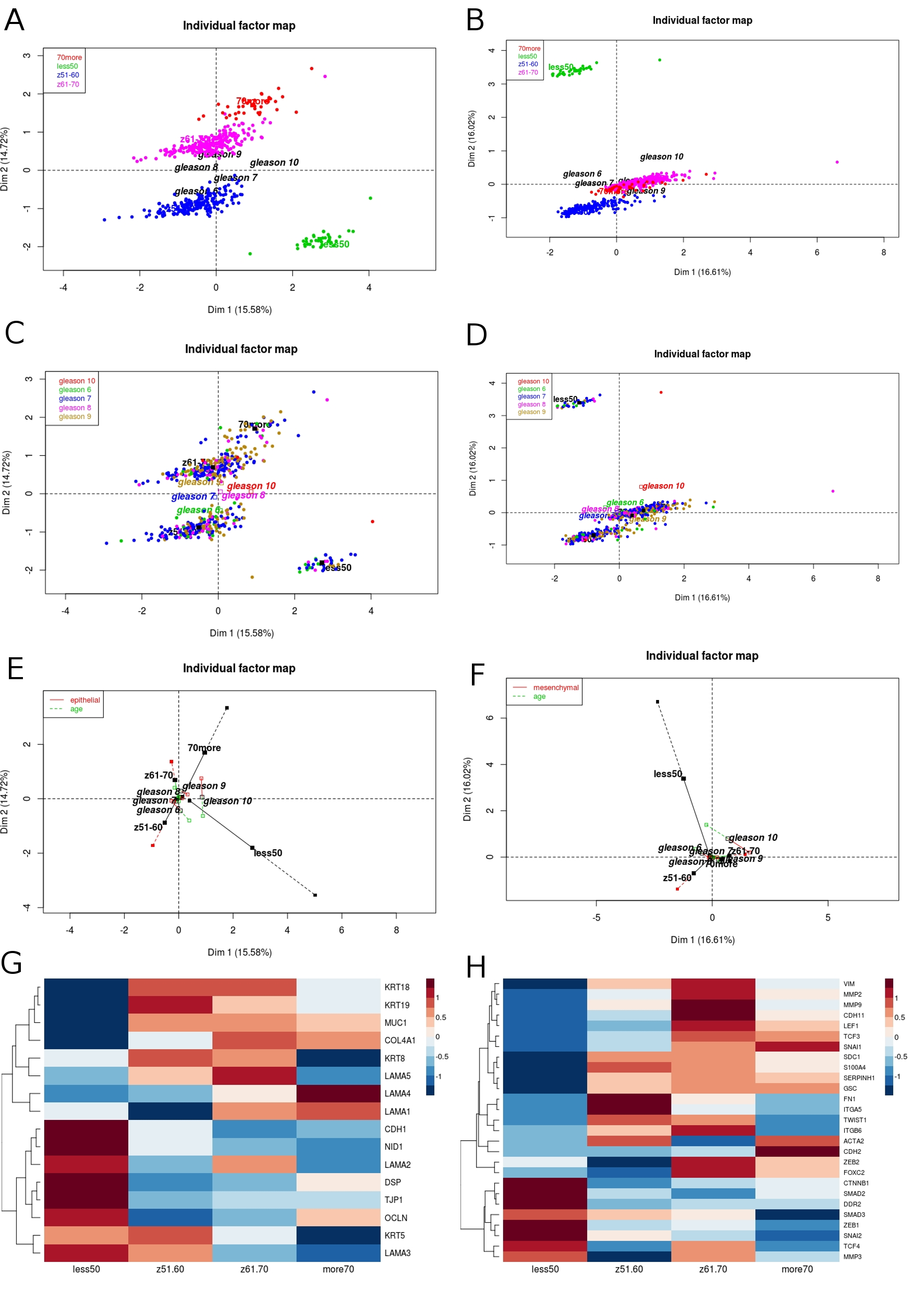

Supplement: S3 Fig — The expression of epithelial (A) vs mesenchymal (B) state markers in separate partitions PRAD patients oppositely. The projections representing contribution of epithelial (C) and mesenchymal (D) state markers along the dimensions indicate significant shit in the EMT model in the age groups, especially in the youngest and the oldest men. In addition, the shift towards more aggressive mesenchymal character of the tumor is observable in the contrasting expression of particular genes involved in the EMT (E) epithelial state markers, F) mesenchymal state markers). (TIFF) [file pone.0188842.s003.tiff]

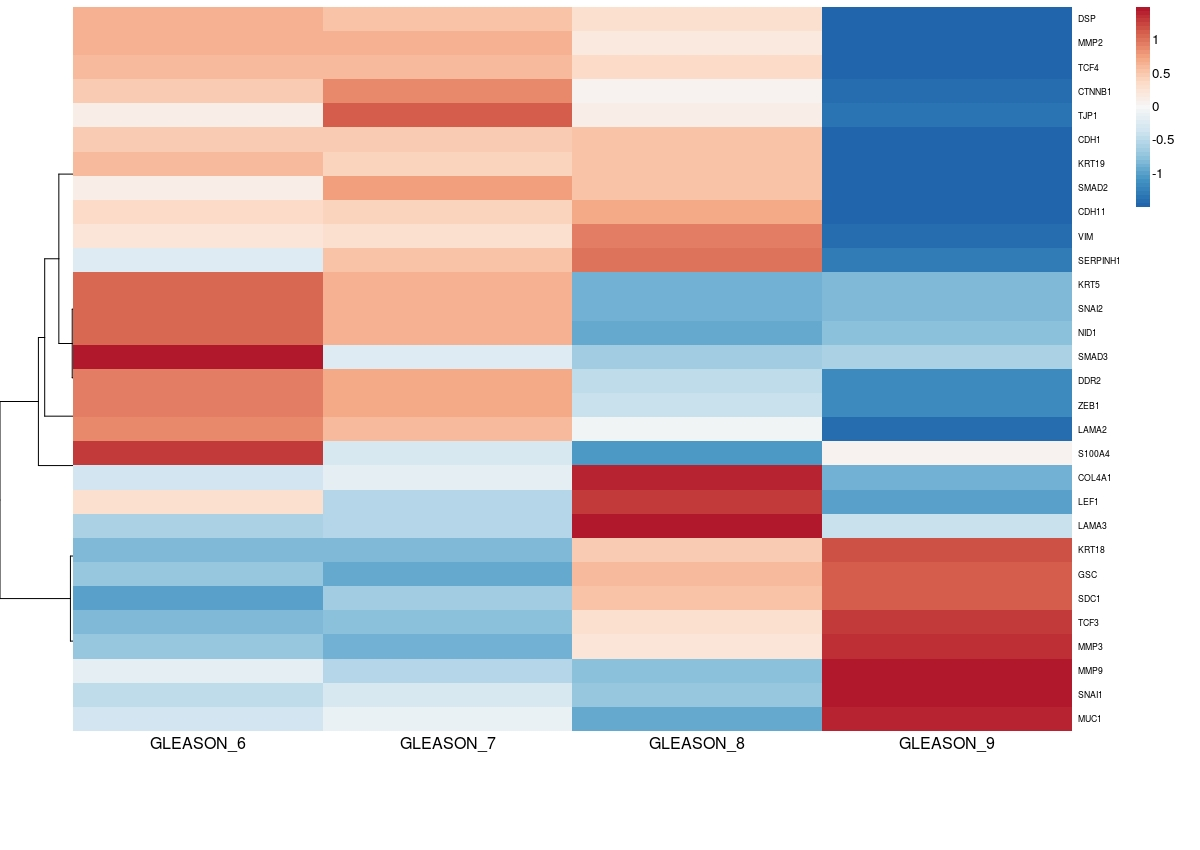

Supplement: S4 Fig — (TIFF) [file pone.0188842.s004.tiff]
